# Identification of a metabolic gene panel to predict the prognosis of myelodysplastic syndrome

**DOI:** 10.1111/jcmm.15283

**Published:** 2020-04-26

**Authors:** Fang Hu, Si‐liang Chen, Yu‐jun Dai, Yun Wang, Zhe‐yuan Qin, Huan Li, Ling‐ling Shu, Jin‐yuan Li, Han‐ying Huang, Yang Liang

**Affiliations:** ^1^ Department of Hematologic Oncology Sun Yat‐Sen University Cancer Center Guangzhou China; ^2^ State Key Laboratory of Oncology in South China Guangzhou China; ^3^ Collaborative Innovation Center for Cancer Medicine Guangzhou China

**Keywords:** gene set enrichment analyses, metabolism, myelodysplastic syndrome, prognostic model, the least absolute shrinkage and selection operator

## Abstract

Myelodysplastic syndrome (MDS) is clonal disease featured by ineffective haematopoiesis and potential progression into acute myeloid leukaemia (AML). At present, the risk stratification and prognosis of MDS need to be further optimized. A prognostic model was constructed by the least absolute shrinkage and selection operator (LASSO) regression analysis for MDS patients based on the identified metabolic gene panel in training cohort, followed by external validation in an independent cohort. The patients with lower risk had better prognosis than patients with higher risk. The constructed model was verified as an independent prognostic factor for MDS patients with hazard ratios of 3.721 (1.814‐7.630) and 2.047 (1.013‐4.138) in the training cohort and validation cohort, respectively. The AUC of 3‐year overall survival was 0.846 and 0.743 in the training cohort and validation cohort, respectively. The high‐risk score was significantly related to other clinical prognostic characteristics, including higher bone marrow blast cells and lower absolute neutrophil count. Moreover, gene set enrichment analyses (GSEA) showed several significantly enriched pathways, with potential indication of the pathogenesis. In this study, we identified a novel stable metabolic panel, which might not only reveal the dysregulated metabolic microenvironment, but can be used to predict the prognosis of MDS.

## 1 BACKGROUND

Myelodysplastic syndromes (MDS) are a heterogeneous clonal haematopoietic disease of haematopoietic stem cells (HSCs) and bone marrow (BM) microenvironment^1^ that mainly occurs in the elderly. MDS is clinical featured by ineffective and dysplastic haematopoiesis, peripheral blood cytopenia and potential of progression into acute myeloid leukaemia (AML) in a third of patients.^2^ MDS was defined by genetic features, morphologic and clinical alterations shared by related myeloid diseases. The pathogenesis of MDS involves gene mutations affecting proliferation, epigenetic modifications, excessive apoptosis of maturing cells, chromosomal abnormalities and a pro‐inflammatory of bone marrow microenvironment and so on.^3^, ^4^ At present, the MDS Revised International Prognostic Scoring System (IPSS‐R) is one of the gold standards for risk stratification and prognostic assessment in MDS patients, in which, patients are categorized into five well‐defined risk groups according to platelet count, haemoglobin levels, absolute neutrophil count (ANC), marrow blast percentage and cytogenetics.^5^, ^6^ Although patients in intermediate‐risk group are reported to have an intermediary survival, it is possible that the disease course might vary, with variable outcome actually.^7^ In the meantime, MDS lacks a diversified prognostic classification system at present. Therefore, identification of more diversified prognostic models would better guide therapeutic decisions, further assisting to design more perfect clinical trials.

Furthermore, MDS is a stem cell‐derived disorder affecting multiple lineages.^8^ MDS stem cells with CD123^+^ have been reported to have higher levels of protein synthesis and change cellular energy metabolism,^9^ which are similar with AML.^10^, ^11^ The anti‐leukaemia mechanism of B cell lymphoma 2 (BCL‐2) inhibitor (venetoclax) combined with demethylated drugs (azacytidine) is the eradication of LSCs by disrupting the tricarboxylic acid (TCA) cycle for further and durable remissions for older AML patients.^12^ Moreover, isocitrate dehydrogenase 2 (IDH2) enzyme inhibitor has been approved by US Food and Drug Administration (FDA) in 2017 for refractory or relapsed AML patients by targeting tumour energy metabolism for. BM microenvironment is vitally involved in the pathogenesis of MDS according to the ‘seed soil’ theory, which consists of cellular components (haematopoietic cells and stromal cells at various stages) and non‐cellular components (metabolites, cytokines, hormones and angiogenic factors).^13^ Leukaemia cells use oxidative phosphorylation for survival, while HSCs depend on glycolysis for energy production.^12^ Leukaemia cells are likely to uptake mitochondria from stromal cells by endocytosis.^14^ As a consequence, metabolism plays key roles for non‐cellular components. Accumulative studies have revealed that the relationship between pathogenesis, treatment and metabolism of MDS recently. Therefore, we established a prognostic panel of metabolic gene by downloading data from Gene Expression Omnibus (GEO) datasets in the training cohort, which was further validated in an independent external cohort. In conclusion, we constructed a metabolic panel to predict the prognosis of MDS and revealed that metabolism played significant roles in the prognosis of MDS.

## 2 MATERIALS AND METHODS

### 2.1 Data collection

The mRNA expression profiles and relevant clinical information were downloaded from GSE58831 ^15^ and GSE114922 ^16^ datasets from the GEO database. The metabolic gene sets utilized as the candidate metabolic gene lists were retrieved from ‘c2.cp.kegg.v7.0.symbols’ in gene set enrichment analysis (GSEA). In addition, perl scripts were used to retrieve metabolic genes for further analysis.

### 2.2 Identification of differentially expressed (DE) mRNA in MDS

Transcripts per million normalization and log2 transformation were performed on the expression profiles. DE analysis was conducted on 861 annotated metabolic‐related genes with protein coding functions by the Limma.^17^ The expression pattern of metabolic genes was examined in training cohort. Genes were subjected to prognostic analysis in the case of consistent expression pattern in training cohort and independent external cohort.

### 2.3 Establishment of the prognostic metabolic gene panel

GSE53381 dataset was used as the training cohort to construct metabolic risk panel. The LASSO regression penalizes the data fitting criteria in a way that eliminates less informative predictor variables to yield simpler and more interpretable models. Therefore, the metabolic panel was constructed according to the penalized maximum likelihood estimator with 1000‐fold cross‐validation. The least criteria of the penalized maximum likelihood estimator were employed to determine the optimal values of penalty parameter λ. In addition, GSE114922 dataset served as an independent external validation cohort. The unified formula determined in the training cohort was used to generate the metabolic risk score in every patient, who were further categorized into high‐ and low‐risk groups according to the median metabolic risk score.

### 2.4 Independence of the prognostic panel

Univariate and multivariate forwarding stepwise Cox regression analyses were conducted in both training and validation cohorts. A *P* < .05 indicated statistical significance.

### 2.5 GSEA

GSEA v4.0.2 software (http://software.broadinstitute.org/gsea/login.jsp) was utilized to recognize the potential biological pathways between high‐ and low‐risk groups by using ‘c2.cp.kegg.v7.0.symbols’ gene sets. NOM *P*‐value < .05 indicated statistical significance and was further exhibited.

### 2.6 Statistical analysis

Time‐dependent receiver operating characteristic (ROC) curve was performed to assess the predictive performance of metabolic signature in the raining and validating cohorts, followed by calculation of area under the curve (AUC) using survival ROC package. Overall survival (OS) was defined as the primary outcome, which was calculated as the date of the study entry until death due to all causes. Kaplan‐Meier curve was plotted by ‘survival’ package, followed by comparison by log‐rank test. Univariable and multivariable Cox analyses were used to evaluate the prognostic performance of clinical and genetic features. Categorical variables were compared by chi‐square test or Fisher's exact test. SPSS^®^ version 24.0 (IBM) and R software (version 3.6.0) were used for statistical analysis. A two sided *P* < .05 indicated statistical significance.

## 3 RESULT

### 3.1 Patient characteristics

Two MDS cohorts involving of 258 patients with gene expression data and clinical data were utilized in the analysis. In brief, GSE58831 dataset was used as the training cohort to establish the prognostic metabolic panel, and patients in GSE114922 cohort served as the external validation cohort for metabolic panel. The median age of patients from GSE58831 and GSE114922 cohorts was 65.23 [range: 19‐87] years and 65.5 [range: 26‐87] years, respectively. The detailed patient characteristics of the two included cohorts were shown in Table 1.

**Table 1:** The detailed patient characteristics of the two included cohorts and the correlation between clinicopathological features and metabolic risk level in training cohort and external validation cohort in MDS

Characteristics	Training cohort		*P‐*value	Validating cohort		*P*‐value
Risk	High risk	Low risk		High risk	Low risk	
Patient	59	60		50	32	
Gender						
Male	47 (79.66%)	31 (51.67%)	<.01	30 (60%)	17 (53.13%)	.54
Age						
>65 y	34 (57.63%)	29 (48.33%)	.31	28 (56%)	18 (56.25%)	.98
WHO_category						
AML‐MDS	2 (3.39%)	2 (3.33%)				
CMML	2 (3.39%)	3 (5.00%)		3 (6%)	1 (3.13%)	
RA	1 (1.70%)	6 (10%)		9 (18%)	10 (31.25%)	
RAEB	8 (13.56%)	4 (6.67%)				
RAEB 1	8 (13.56%)	6 (10%)		12 (24%)	4 (12.5%)	
RAEB 2	17 (28.81%)	/		9 (18%)	5 (15.63%)	
RARS‐T	/	4 (6.67%)				
RARS	4 (6.78%)	7 (11.67%)		17 (34%)	12 (37.50%)	
RCMD	9 (15.25%)	11 (18.33%)				
RCMD‐RS	8 (13.56%)	11 (18.33%)				
5q‐	/	6 (10%)				
Karyotype						
Normal	15 (25.42%)	19 (31.67%)	.45	32 (64%)	18 (56.25%)	.48
Non‐normal	44 (74.58%)	41 (68.33%)		18 (36%)	14 (43.75%)	
IPSS						
High	5 (8.47%)	1 (1.67%)	<.01	1 (2%)	2 (6.25%)	.33
int‐1	21 (35.60%)	29 (48.33%)		21 (42%)	15 (46.89%)	
int‐2	16 (27.12%)	4 (6.67%)		11 (22%)	2 (6.25%)	
Low	12 (20.34%)	22 (36.67%)		14 (28%)	12 (37.50%)	
Transfusion dependent						
Dependent	30 (50.85%)	18 (30.00%)	.06	18 (36%)	7 (21.88%)	.25
Independent	28 (47.46%)	35 (58.33%)		31 (62%)	22 (68.75%)	
Haemoglobin (mg/dL)						
≤80	9 (15.25%)	10 (16.67%)	.77	10 (20%)	2 (6.25%)	.1
>80	49 (83.05%)	47 (78.33)		39 (78%)	28 (87.5%)	
Blasts cells in BM (%)						
≤10	35 (59.32%)	45 (75.00%)	.03	40 (80%)	25 (78.13%)	.71
<10<20	17 (28.81%)	8 (13.33%)		10 (20%)	5 (15.63%)	
Platelet count (×10^9^/L)						
≤40	8 (13.56%)	3 (5.00%)	.12	5 (10%)	1 (3.13%)	.27
>40	50 (84.75%)	54 (90%)		45 (90%)	29 (90.63%)	
Absoulte neutrophile count (×10^9^/L)						
≤1.8	33 (55.93%)	22 (36.67%)	.05	25 (50.00%)	8 (25.00%)	.03
	23 (38.98%)	33 (55.00%)		23 (46.00%)	22 (68.75%)	

### 3.2 Establishment and validation of the prognostic metabolic gene panel

**Figure 1:**
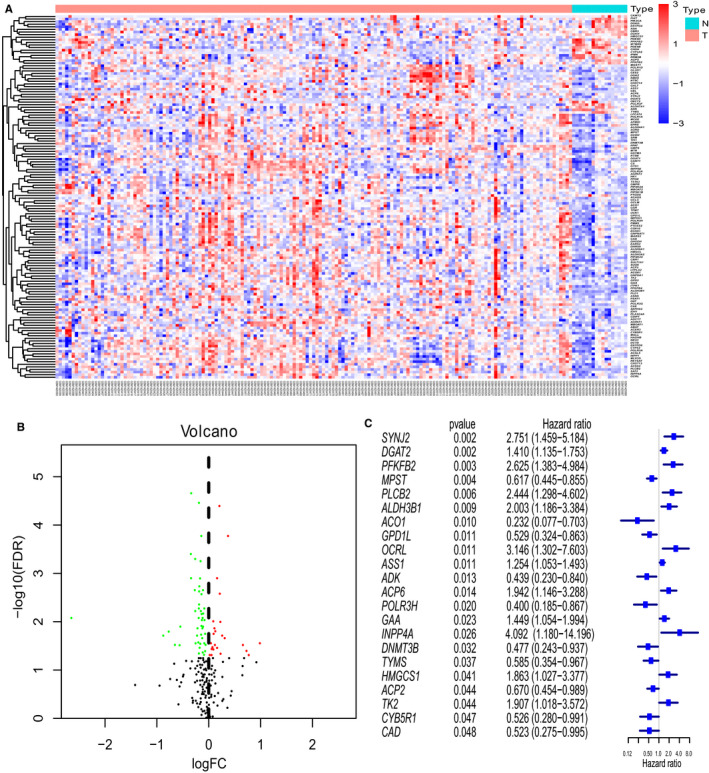
Identification of metabolic gene panel (A) Heat map of differential expressed genes (DEGs) between MDS patients and healthy individuals (Padj < 0.05). B, Volcano plot of DEGs. C, Univariate Cox regression identified 22 survival‐related genes

**Figure 2:**
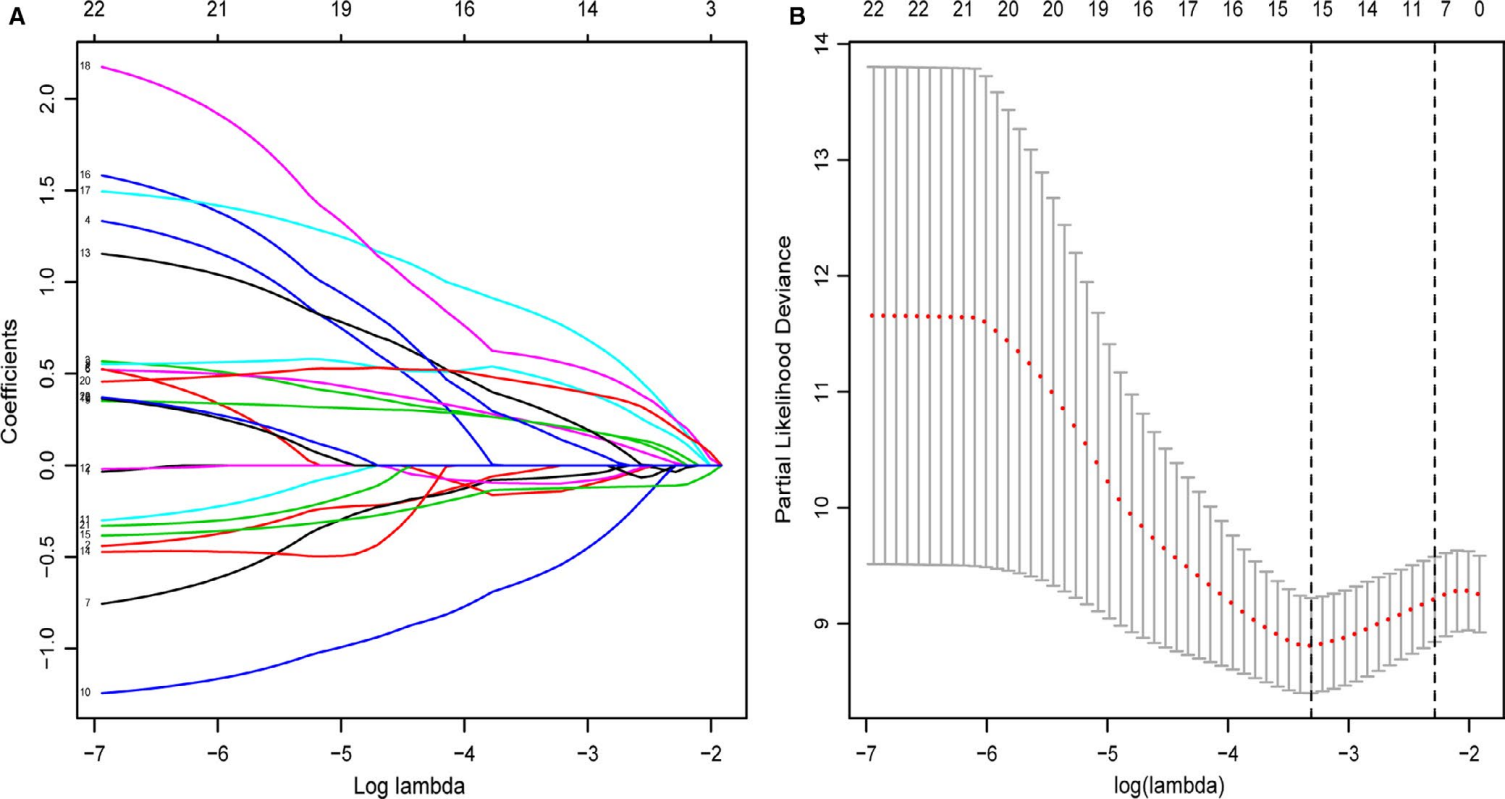
Construction of the prognostic model for MDS (A) LASSO coefficients of metabolism‐related genes. Each curve represents a metabolic gene. (B) 1000‐fold cross‐validation to select variants in the LASSO regression via min criteria

**Figure 3:**
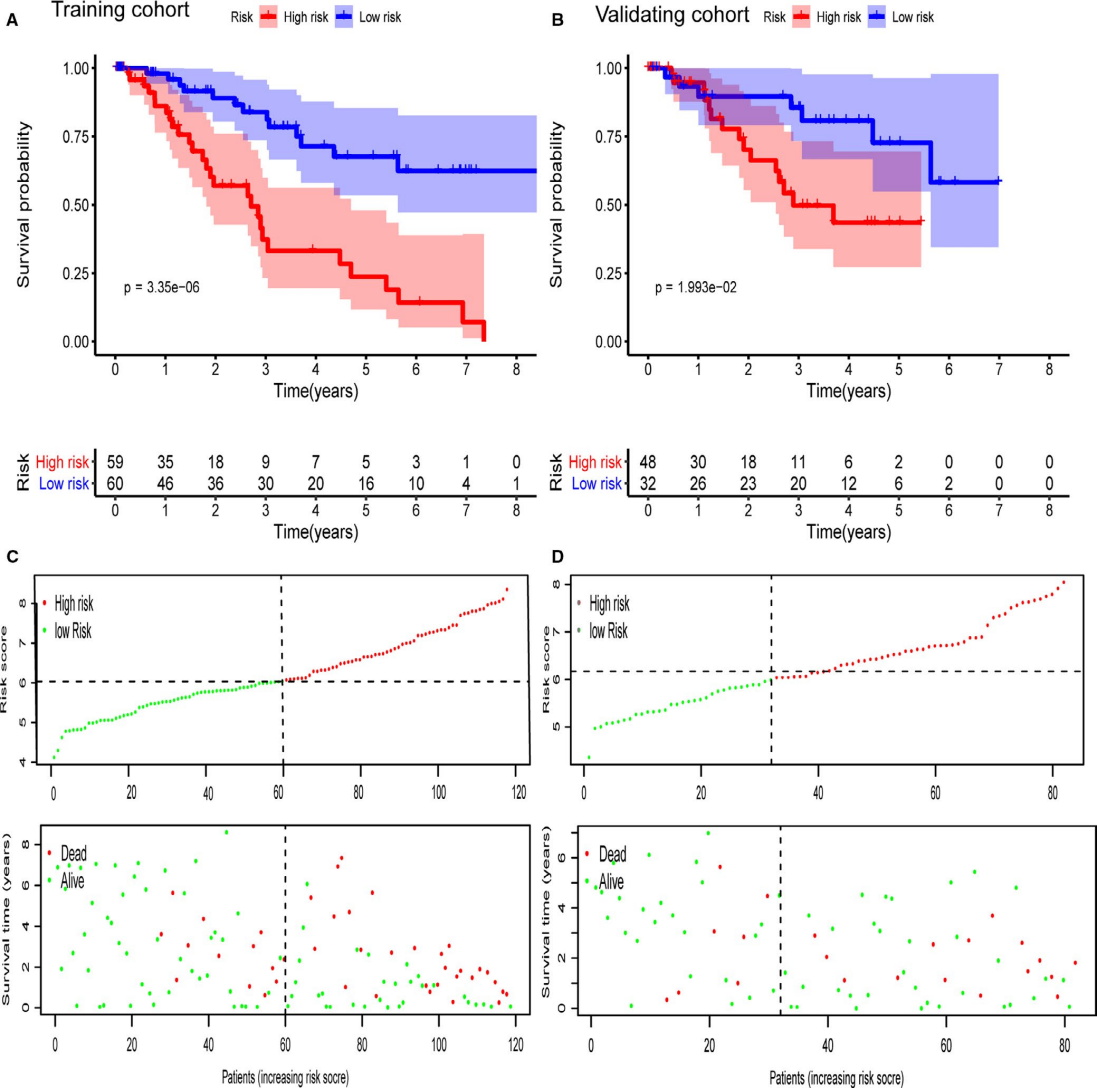
Kaplan–Meier (KM) analysis, risk score analysis for the 15‐gene panel in MDS (A) KM curve of the fifteen‐gene panel in the training cohort. B, KM curve of the 15‐gene panel in the validation cohort. C, Risk score analysis of the 15‐gene panel in the training cohort. D, Risk score analysis of the 15‐gene panel in the validation cohort

**Figure 4:**
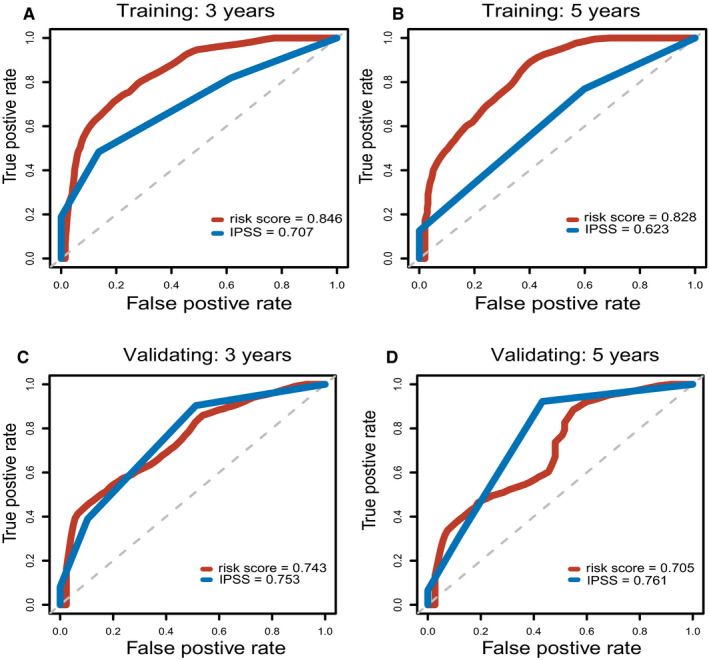
Time‐dependent ROC analysis for the 15‐gene panel in MDS Time‐dependent ROC analysis for (A) 3‐year OS and (B) 5‐year OS of the 15 gene panel in the training cohort. Time‐dependent ROC analysis for (C) 3‐year OS and (D) 5‐year OS of the 15 gene panel in the validating cohort

Among the 861 metabolic genes subjected to DE analysis by the Limma, 140 genes were differently expressed between healthy sample and MDS sample (Figure 1A,B). Further, the prognostic values of these 140 genes were analysed via Univariate Cox regression analysis. Ultimately, 22 genes that were differentially expressed as well as survival‐related (*P* < .05) were identified. (Figure 1C). Afterwards, the Lasso‐penalized Cox analysis regression was used to select the most useful predictive genes from the 22 genes. A penalized maximum likelihood estimator was performed with 1000 bootstrap replicates. The regularization parameter lambda was used to identify the optimal weighting coefficients via the least criteria (Figure 2A,B). Afterwards, 15 metabolic genes were selected and the coefficient was estimated to construct the metabolic prognostic model. The 15 metabolic genes included in the prognostic model were exhibited Table 2. The MDS patients were categorized into high‐ and low‐risk group according to the median of risk score. The prognosis was significantly different between high‐ and low‐risk groups, and the survival was poorer in patients from the high‐risk group than those from the low‐risk group (*P* < .01; Figure 3A‐D). The 5‐year OS rates of high‐risk and low‐risk groups were 23.7% [95%CI (0.12, 0.48)] and 67.6% [95%CI (0.54, 0.85)], respectively. The 3‐ and 5‐year AUC of OS was 0.846 and 0.828 in GSE58831, respectively (Figure 4A,B). The metabolic prognostic panel showed better prognostic predictive ability compared to the known IPSS scoring system.

**Table 2:** A 15‐gene panel signature identified by Lasso Cox regression analysis

Gene	Coef	Metabolic‐related KEGG pathways
*ACP2* (Acid Phosphatase 2, Lysosomal)	−0.00046	Riboflavin metabolism
*ACP6* (Acid Phosphatase 6, Lysophosphatidic)	0.211033	Phospholipid metabolism
*ALDH3B1* (Aldehyde Dehydrogenase 3 Family Member B1)	0.447464	Beta‐Alanine metabolism; Histidine metabolism
*ASS1* (Argininosuccinate Synthase 1)	0.200934	Alanine, aspartate and glutamate metabolism
*CAD* (Carbamoyl‐Phosphate Synthetase 2, Aspartate Transcarbamylase And Dihydroorotase)	−0.06062	Alanine, aspartate and glutamate metabolism; Pyrimidine metabolism
*CYB5R1* (Cytochrome B5 Reductase 1)	−0.14185	Amino sugar and nucleotide sugar metabolism
*DGAT2* (Diacylglycerol O‐Acyltransferase 2)	0.21335	Glycerolipid metabolism
*DNMT3B* (DNA Methyltransferase 3 Beta)	−0.54252	Cysteine and methionine metabolism
*GPD1L* (Glycerol‐3‐Phosphate Dehydrogenase 1 Like)	−0.09914	Glycerophospholipid metabolism
*HMGCS1* (3‐Hydroxy‐3‐Methylglutaryl‐CoA Synthase 1)	0.260982	Butanoate metabolism
*MPST *(Mercaptopyruvate Sulfurtransferase)	−0.12544	Cysteine and methionine metabolism; Metabolic pathways
*OCRL* (OCRL Inositol Polyphosphate‐5‐Phosphatase)	0.140246	Inositol phosphate metabolism
*PFKFB2* (6‐Phosphofructo‐2‐Kinase/Fructose‐2,6‐Biphosphatase 2)	0.767954	Fructose and mannose metabolism
*PLCB2* (Phospholipase C Beta 2)	0.561048	Inositol phosphate metabolism
*SYNJ2* (Synaptojanin 2)	0.419988	Inositol phosphate metabolism

The metabolic prognostic model was subsequently validated in the external cohort of GSE114922. Similarly, patients were divided into low‐ and high‐risk groups based on the median risk score from the training cohort. Consistent with the training set, patients from the low‐risk group had favourable outcome. The 5‐years OS rates of patients from high‐risk and low‐risk groups were 43.5% [95%CI (0.27, 0.69)] and 72.7% [95%CI (0.55, 0.96)], respectively. The AUC of 3‐ and 5‐year OS was 0.743 and 0.705 in the validation cohort, respectively (Figure 4C,D). Consistently, the metabolic prognostic panel showed comparative prognostic predictive ability in the validation external cohort in comparison with the known IPSS scoring system. Nevertheless, it is better to validate in a large sample size.

### 3.3 Independent prognostic role of the metabolic gene panel

**Figure 5:**
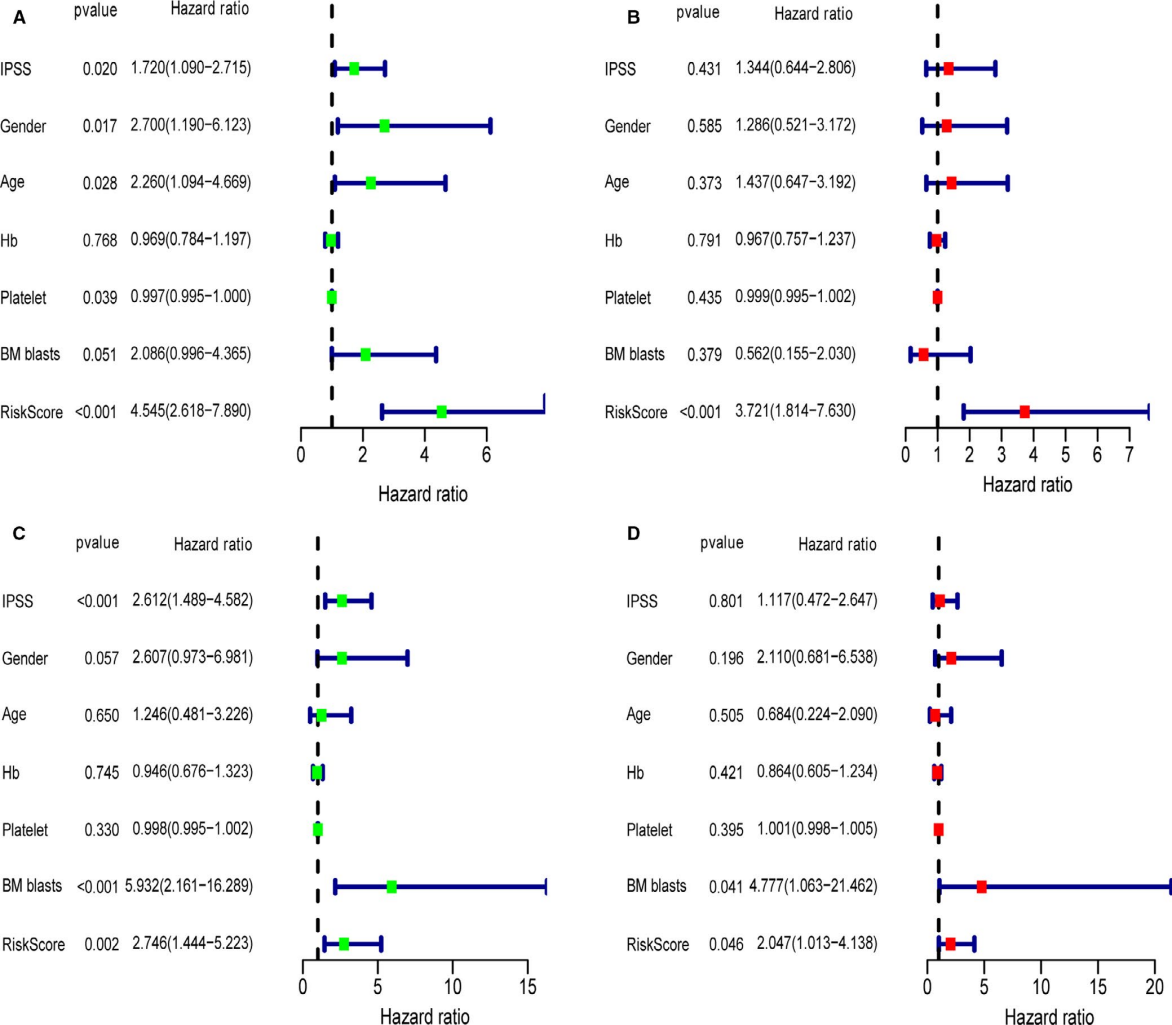
Forrest plot of the univariate and multivariate Cox regression analysis in MDS. Forrest plot of the (A) univariate and (B) multivariate Cox regression analysis in the training cohort. Forrest plot of the (C) univariate and (D) multivariate Cox regression analysis in the validating cohort

The univariate Cox analysis for risk score and other clinical clinicopathological showed there was significant association of OS of MDS patients with gender, IPSS, the risk score, age, platelet in the training cohort. Further multivariate analysis showed that the risk score was still an independent predictive factor (HR: 3.721, 95%CI: 1.814‐7.630) after adjusting clinical covariates (Figure 5A,B). Furthermore, the risk score remained as an independent predictor (HR: 2.047, 95%CI: 1.013‐4.138) after adjusting clinical covariates in the external validating cohort (Figure 5C,D).

### 3.4 Association between the metabolic risk level and clinicopathological features

**Figure 6:**
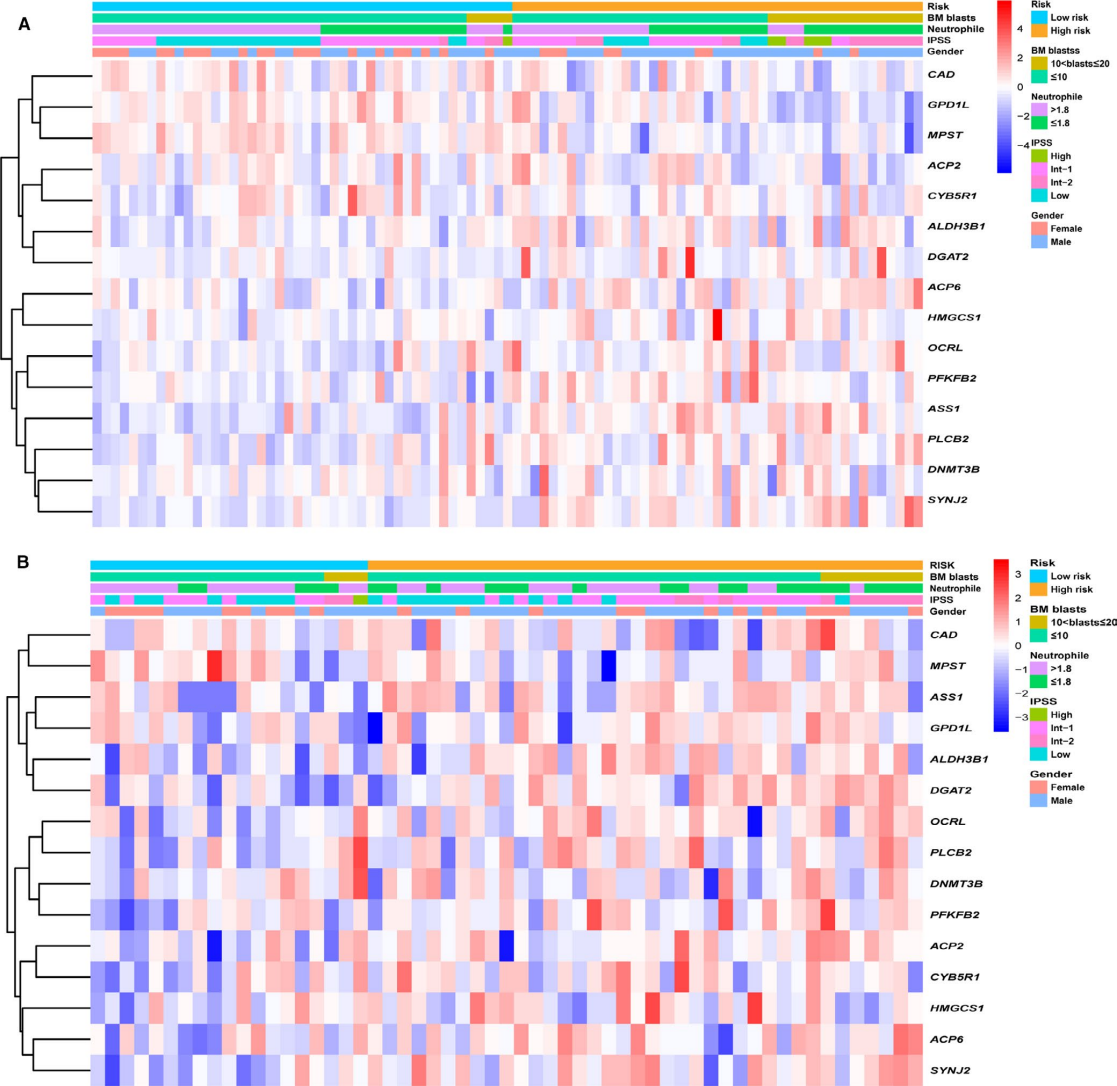
Heat map of the expression of 15 metabolic gene panel and clinicopathological characteristics in different metabolic risk group for the (A) training cohort and (B) validation cohort

In total, 201 patients with complete clinical data including age, gender, WHO category, karyotype, IPSS, transfusion dependent, haemoglobin, bone marrow blasts cells, platelet count and absolute neutrophil count were included in the training and validation cohort. High‐risk patients were associated with male, higher numbers of bone marrow blast cells, higher IPSS score and lower absolute neutrophil count (Table 1). However, there are no significant statistical difference of clinical character except for the absolute neutrophile count in different metabolic risk level of validating cohort. The small sample size is a possible reason. The distribution of clinical characteristics and gene expression in different metabolic risk group was visualized in Figure 6A,B.

### 3.5 GSEA

**Figure 7:**
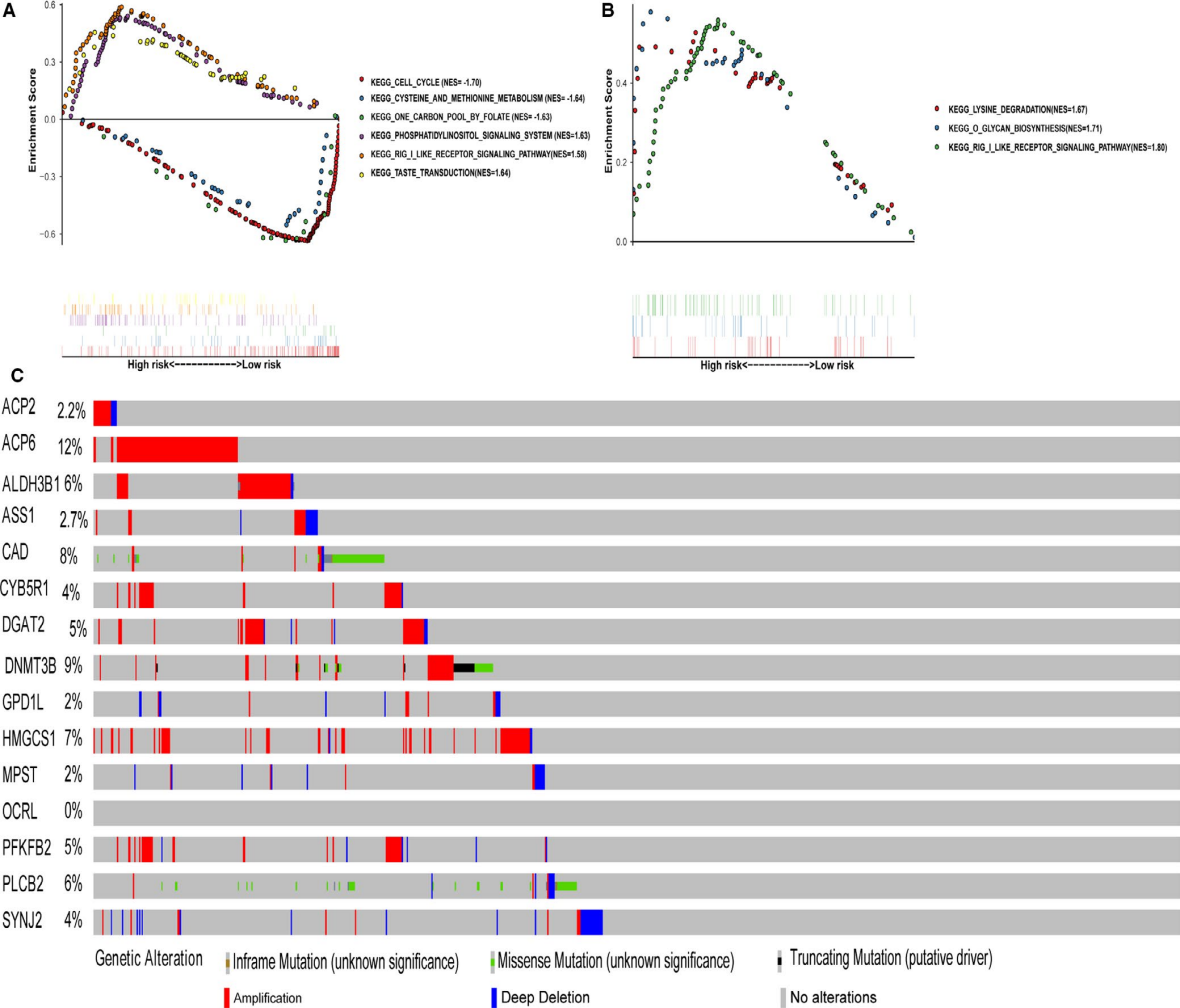
The significantly enriched KEGG pathways by GSEA; Genetic alterations of the 15 genes in Broad Institute Cancer Cell Line Encyclopedia CCLE. Representative Top 3 enriched KEGG pathways in the (A) training cohort and (B) validation cohort. C, Genetic alterations of the 15‐gene panel in CCLE, obtained from the cBioportal for Cancer Genomics (http://www.cbioportal.org/)

GSEA identified 36 significantly enriched KEGG pathways in the training or validation cohort. The majority of the metabolism‐associated pathways were enriched in the low‐risk group, and the metabolic pathways ranked by NES were cysteine and methionine metabolism, glycine serine and threonine metabolism, fatty acid metabolism and pyrimidine metabolism. On the contrary, the majority of the non‐metabolism‐associated pathways were enriched in the high‐risk group. Additionally, most enriched pathways were correlated with cancer (such as the cell cycle and phosphatidylinositol signalling system) or metabolism (such as the glycine serine and threonine metabolism, cysteine and methionine metabolism) (Figure 7A,B).

### 3.6 External validation using online database

The mutation variants of the metabolic gene panel were explored in CCLE database by the cBioportal for Cancer Genomics.^18^, ^19^ As was expected, the gene amplification, which can change gene expression, was the most common alteration form of this metabolic genes. Meanwhile, *ACP6*, *ALDH3B1*, *CYB5R1*, *DGAT2*, *DNMT3B*, *HMGCS1* and *PFKFB2* possessed the most frequently genetic alterations in the metabolic gene panel (Figure 7C). But no *OCRL* mutations have been reported. Meanwhile, gene amplification of *GPD1L*, *MPST*, *PLCB2*, *SYNJ2* accounted for a small proportion of total mutations. Taking together, further validation of the aberrant expression of the metabolic gene panel was performed in cell lines, which revealed that the abnormal expression of these genes might be due to genetic alteration to some extent. The cBioPortal for Cancer Genomics further was used to analyse the relationship of expression and mutation of the metabolic gene in Cancer Cell Line Encyclopedia (CCLE) samples (Figure S1), which further verified the potential mechanism of the expressed variety of 15 metabolic gene.

## 4 DISCUSSION

Previous studies have revealed the reprogramming of glucose metabolism in multiple types of malignant tumour. To provide biosynthetic precursors or energy, glycolysis is accelerated in malignant cells. Meanwhile, the active truncated TCA cycle also generates intermediates for cancer cells.^20^ Patients with low‐risk MDS exhibit an ido/tph1 enzyme activity imbalance which regulates tryptophan by indoleamine 2,3‐dioxygenase (IDO) and tryptophan 2,3‐dioxygenase (TDO).^21^ A previous study has revealed that excessive weight gain would increase MDS risk by fetuin‐A, adiponectin and free leptin.^22^ Therefore, the metabolic‐related pathway and genes not only play significant roles in pathogenesis of MDS, but also impact prognosis in patients. However, there is a lack of metabolism‐related MDS model for prognostic prediction. To this end, we constructed a metabolic gene panel based on the identified metabolic to predict prognosis in MDS patients.

In the present study, a novel 15‐gene metabolic panel model was constructed based on data from the training cohort, which was further validated in the validation cohort of GSE114922 dataset. The model could divide patients into high‐risk (with poor prognosis) and low‐risk (with favourable prognosis) groups. Furthermore, patients in the high‐risk group were related with poor clincopathological factors. In addition, the reliability and stability of the prognostic model were further confirmed in both cohorts. The metabolic panel model showed comparable or even better prognostic performance compared with IPSS prognostic stratification.

Fifteen metabolic genes were identified to construct the metabolic model, most of which have been reported to be involved in malignancy. *PFKFB2*, a vital regulator of glucose metabolism, has been defined as a candidate gene for GC‐triggered apoptosis according to comparative expression profiling in childhood acute lymphoblastic leukaemia (ALL).^23^ Interestingly, *PFKFB2* was suppressed by miR‐613 in gastric cancer, which could further inhibit cell proliferation and invasion.^24^ The expression pattern is reported for the first time as a potential marker in MDS *PLCB2*, involved in inositol phosphate metabolism, has been narrowly linked to the poor prognosis in patients with hepatocellular carcinoma, lung cancer and mammary carcinoma.^25^ In our study, *PLCB2* was negatively correlated with the prognosis of MDS *Dnmt3b* has been previously reported to suppress Myc‐induced lymphomagenesis in a mouse model, while loss of *Dnmt3b* accelerates *MLL‐AF9* leukaemia progression via enhancing stemness and promoting cell cycle progression,^26^, ^27^, ^28^ which are consistent with our conclusion. *ALDH3B1*, involved in phenylalanine metabolism, is dynamically modulated during myelopoiesis, with up‐regulated expression in mature granulocytes in mice and in promyelocytes in humans, and down‐regulated expression during myeloid maturation.^29^ *ASS1*, an argininosuccinate synthetase, is heterogeneously expressed in AML populations.^30^ At present, arginine deprivation has been shown to kill tumour cells but not normal cells, with numerous undergoing clinical trials of arginine deprivation.^31^, ^32^ *ASS1* levels might suggest the more sensitive candidate to be developed as a biomarker for identification of AML samples which might be sensitive to arginine deprivation.^33^ Currently, the roles of the metabolic genes in pathogenesis of MDS should be further explored.

The results of GSEA revealed that there were multiple significantly enriched pathways. Interestingly, patients in the low‐risk group were related to the metabolic pathways, while patients in the high‐risk group were associated with phosphatidylinositol and RIG I like receptor signalling pathway. And the phosphatidylinositol signal system was involved in cell growth regulation. As a consequence, the dysregulated metabolic‐related signalling pathways might shed novel light on the treatment of MDS. As is reported that the gene expression can be altered by genomic copy number gains, losses and other mutations.^34^≥12% of alterations in gene expression is attributable to the variations of gene copy number,^35^ which may be a potential mechanism of the expressed variation of 15 metabolic genes.

On the one hand, we have established a robust prognostic model based on metabolic gene that complements the existing risk stratification for MDS. On the other hand, several limitations of this study should also be acknowledged. Firstly, we are unavailable to more clinical information due to the data driving from GEO database. Secondly, the significance of the metabolic panel should be further confirmed in real‐world research, and further basic experiments are simultaneously necessary to explore the underlying pathogenesis.

In summary, we constructed a novel prognostic prediction model based on metabolic genes from GEO database for MDS, and further validated in the validation cohort. The prognostic model was not only an independent prognostic predictor for MDS but also reflected the disordered metabolism of MDS. Moreover, this panel could be utilized as an effective approach for prognostic prediction in MDS patients in clinical practice.

## CONFLICT OF INTEREST

The authors declare no conflict of interest.

## AUTHOR CONTRIBUTIONS

Conceptualization, FH and YL; methodology, CSL, and FH; validation, CSL; formal analysis,; investigation, FH, CSL, and DYJ; resources, YL; data curation, CYY; writing—original draft preparation, YW, and FH; writing—review and editing, YW, FH and JYL; visualization, SLC, and LLS and DJD; supervision, YL; project administration, YL; funding acquisition, YL.

## Supporting information

Figure S1

## Data Availability

The GSE58831 and GSE114922 datasets were collected via the Gene Expression Omnibus (GEO) database, which were utilized for retrieving clinicopathological data and RNA expression patterns. All data or code generated or used during this study are available from the corresponding author by reasonable request.
