# Supplementary figures and images for: Identification of a metabolic gene panel to predict the prognosis of myelodysplastic syndrome
Source: J Cell Mol Med. 2020 Apr 26;24(11):6373–84. doi: 10.1111/jcmm.15283 (PMC7294120; doi:10.1111/jcmm.15283)

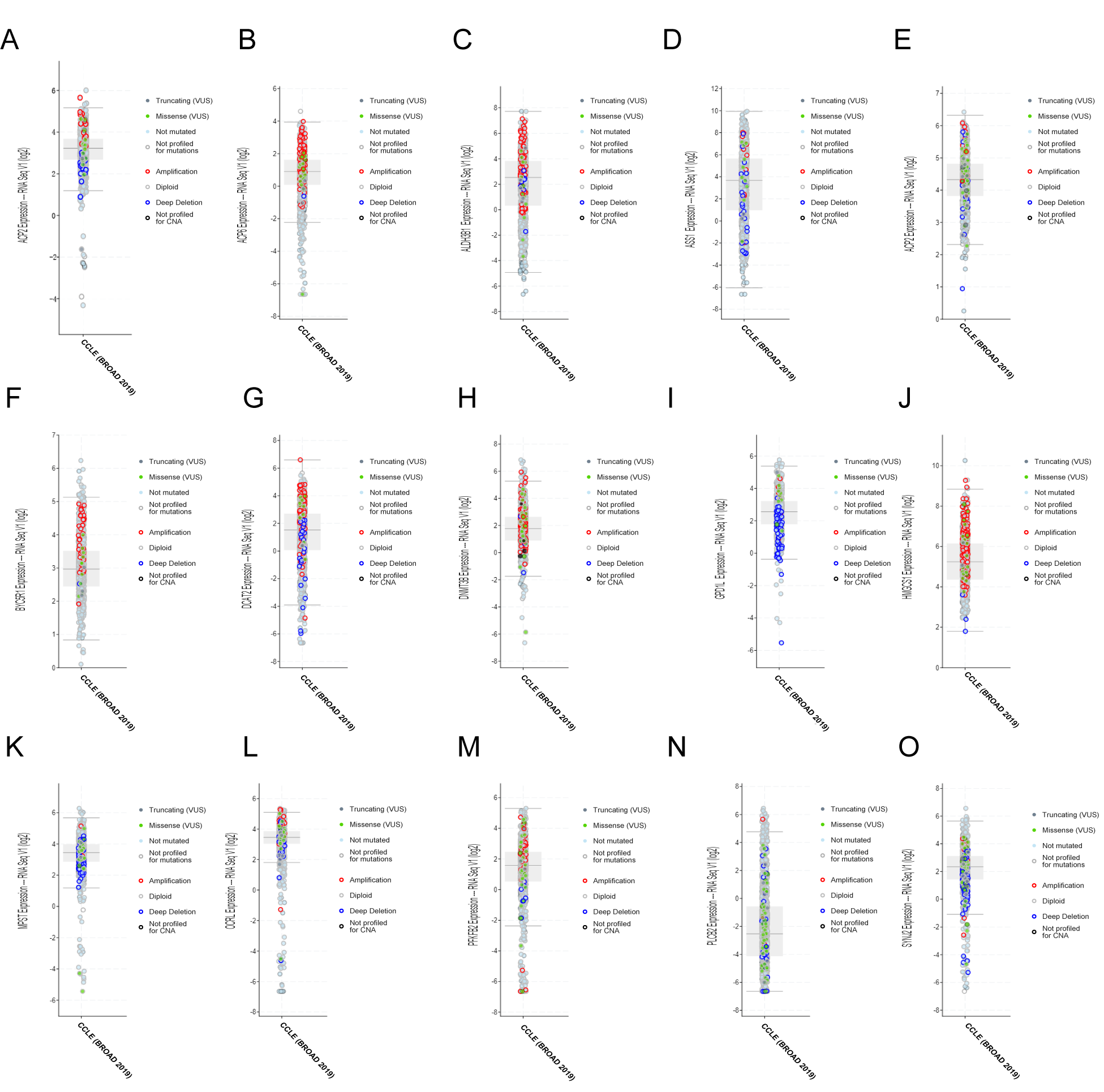

Supplement: Supplementary file 1 — Figure S1 [file JCMM-24-6373-s001.tif]
